# Mediterranean Diet and Cardiodiabesity: A Systematic Review through Evidence-Based Answers to Key Clinical Questions

**DOI:** 10.3390/nu11030655

**Published:** 2019-03-18

**Authors:** Marcella Franquesa, Georgina Pujol-Busquets, Elena García-Fernández, Laura Rico, Laia Shamirian-Pulido, Alicia Aguilar-Martínez, Francesc Xavier Medina, Lluís Serra-Majem, Anna Bach-Faig

**Affiliations:** 1 Faculty of Health Sciences, Universitat Oberta de Catalunya (Open University of Catalonia, UOC), 08018 Barcelona, Spain; mfranquesa@igtp.cat (M.F.); georgipbg@uoc.edu (G.P.-B.); elenagf85@uoc.edu (E.G.-F.); lricoca@uoc.edu (L.R.); lshamirian@uoc.edu (L.S.-P.); 2 REMAR-IVECAT Group, Health Science Research Institute Germans Trias i Pujol, Can Ruti Campus, 08916 Badalona, Spain; 3 Division of Exercise Science and Sports Medicine, Department of Human Biology, Faculty of Health Sciences, University of Cape Town, 7725 Cape Town, South Africa; 4 FoodLab Research Group (2017SGR 83), Faculty of Health Sciences, Universitat Oberta de Catalunya (Open University of Catalonia, UOC), 08018 Barcelona, Spain; aaguilarmart@uoc.edu (A.A.-M.); fxmedina@uoc.edu (F.X.M.); 5 CIBER de Fisiopatología de la Obesidad y la Nutrición (CIBEROBN), Instituto de Salud Carlos III, 28029 Madrid, Spain; lserra@dcc.ulpgc.es; 6 Research Institute of Biomedical and Health Sciences, University of Las Palmas de Gran Canaria, 35001 Las Palmas de Gran Canaria, Spain; 7 Food and Nutrition Area, Barcelona Official College of Pharmacists, 08009 Barcelona, Spain

**Keywords:** Mediterranean Diet, diabetes mellitus, cardiovascular disease, metabolic syndrome, obesity, cardiodiabesity, review, PICO

## Abstract

The Mediterranean Diet (MedDiet) has been promoted as a means of preventing and treating cardiodiabesity. The aim of this study was to answer a number of key clinical questions (CQs) about the role of the MedDiet in cardiodiabesity in order to provide a framework for the development of clinical practice guidelines. A systematic review was conducted to answer five CQs formulated using the Patient, Intervention, Comparison, and Outcome (PICO) criteria. Twenty articles published between September 2013 and July 2016 were included, adding to the 37 articles from the previous review. There is a high level of evidence showing that MedDiet adherence plays a role in the primary and secondary prevention of cardiovascular disease (CVD) and improves health in overweight and obese patients. There is moderate-to-high evidence that the MedDiet prevents increases in weight and waist circumference in non-obese individuals, and improves metabolic syndrome (MetS) and reduces its incidence. Finally, there is moderate evidence that the MedDiet plays primary and secondary roles in the prevention of type 2 diabetes mellitus (T2DM). The MedDiet is effective in preventing obesity and MetS in healthy and at-risk individuals, in reducing mortality risk in overweight or obese individuals, in decreasing the incidence of T2DM and CVD in healthy individuals, and in reducing symptom severity in individuals with T2DM or CVD.

## 1. Introduction

**Figure 1:**
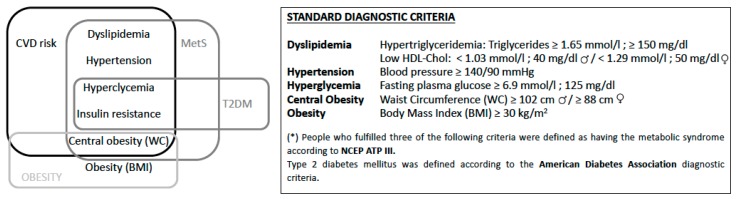
Summary of cardiodiabesity and standard diagnostic criteria. Cardiodiabesity encompasses cardiovascular disease (CVD), type 2 diabetes mellitus (T2DM), metabolic syndrome (MetS), and obesity. Note: Reproduced with permission from García-Fernández et al. [6]. Abbreviations not previously defined: HDL-Chol, high-density lipoprotein cholesterol.

A growing body of scientific evidence shows that the Mediterranean Diet (MedDiet) has a beneficial effect on obesity, metabolic syndrome (MetS), cardiovascular disease (CVD), and type 2 diabetes mellitus (T2DM) [1,2,3,4]. These four diseases are so inherently linked that a new umbrella term, *cardiodiabesity*, has been adopted to reflect their coexistence and interrelationship [5,6] (Figure 1). According to the International Diabetes Federation, T2DM is expected to become the seventh leading cause of death by 2030 [7]. One of the main causes of T2DM is obesity, which is now a worldwide epidemic despite efforts by the World Health Organization to meet the target of a 25% relative reduction in premature mortality from non-communicable diseases [7]. If the current trend continues, by 2025, approximately 18% of men and over 21% of women will be obese, up from the current rates of 10.8% and 14.9%, respectively [8]. The potential rise in the global incidence of cardiodiabesity is alarming, as central obesity and visceral adiposity have already been identified as causative agents of T2DM and CVD [8].

The word *diet* comes from the original Greek term *diaita* (way of living), and the MedDiet [9]—describing traditional dietary and lifestyle habits in the Mediterranean region—has attracted international interest as a healthy, prudent dietary pattern [10] that can, as shown by extensive evidence, contribute to the prevention of chronic diseases [11].

Dietary recommendations can play an important role in the prevention of certain diseases. Not all physicians, however, are willing to offer nutritional advice, as they feel that they lack the necessary knowledge to confidently discuss these issues with their patients [12]. The main reasons for this reluctance include a lack of time or information, the need for cultural adaptations to dietary patterns and guidelines, and the complexity and contradictions of existing recommendations [13,14,15]. Even physicians themselves do not have high levels of MedDiet adherence, probably due in part to away-from-home eating, which is associated with poor health outcomes [15]. Health professionals could benefit from clinical practice guidelines (CPGs), which have been defined as recommendations developed systematically to help professionals and patients make decisions about the most appropriate health care and to select the diagnostic or therapeutic options that are best suited to addressing a health problem or a specific clinical condition [15]. 

According to the Appraisal of Guidelines for Research and Evaluation (AGREE) tool, the first step in drawing up CPGs is to define a clear set of clinical questions (CQs) using the Patient, Intervention, Comparison, and Outcome (PICO) criteria [16,17,18]. The next step is to establish systematic and explicit criteria for reviewing and assessing the available scientific evidence to provide answers to these questions. The aim of this study was to establish a theoretical framework for the development of CPGs on the application of the MedDiet in patients with conditions grouped under the umbrella term *cardiodiabesity*. To do this, existing evidence on the association between MedDiet adherence and collective cardiodiabesity risk was updated [6]. The findings presented in this paper should provide experts with the basis for developing CPGs to promote the provision of evidence-based nutrition information and advice to patients with obesity, MetS, CVD, and T2DM. 

## 2. Materials and Methods

### 2.1. Literature Search

A thorough search of prospective cohort, cross-sectional, and clinical trial studies in the scientific literature was conducted to gather evidence on the ability of the MedDiet to modulate or prevent diseases encompassed by the term *cardiodiabesity*. Using the same search strategies as García-Fernández et al. [6], the available evidence on the association between the MedDiet and cardiodiabesity was updated by reviewing studies published between September 2013 and July 2016. The literature search was performed in PubMed using the search term *Mediterranean Diet* and the key words *Diabetes Mellitus*, *Coronary Disease, Myocardial Ischemia, Heart Disease, Metabolic Syndrome*, and *Obesity*.

### 2.2. Inclusion Criteria

Five CQs were defined using the PICO framework (Table 1) [16,17,18]. As in the earlier review by García-Fernández et al. [6], only those studies relating to T2DM, obesity, MetS, and CVD were eligible for inclusion. In this study, we applied more stringent selection criteria (Table 2) to the articles from the previous study and to the new ones. The studies included were assigned one of three levels of evidence (Table 3) to answer the formulated CQs and to establish recommendations for the CPGs.

**Figure 2:**
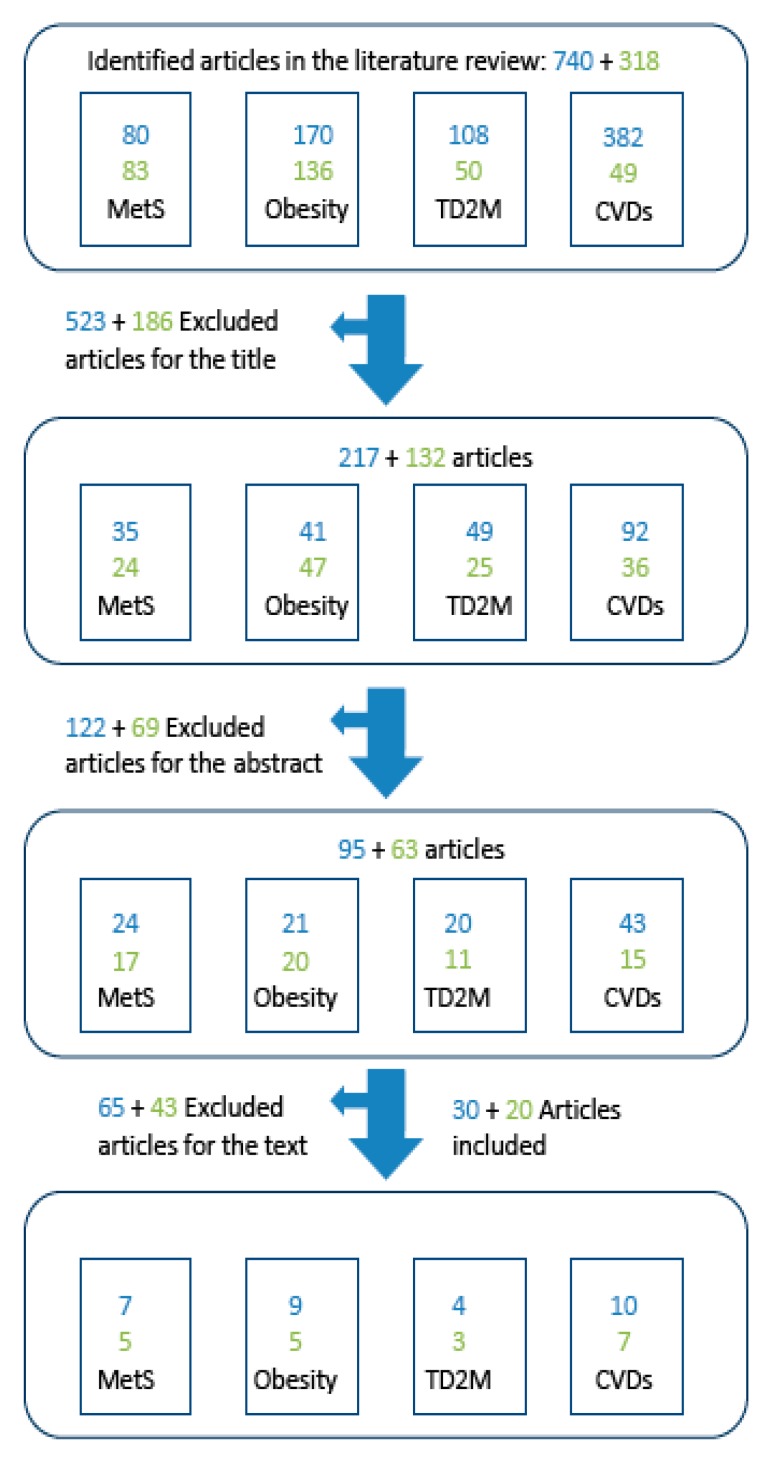
Selection process for studies included in the systematic review. The numbers in blue refer to the articles included and excluded by García-Fernández et al. [6]. The numbers in green refer to the additional articles included in and excluded from this updated review.

The literature search was limited to human studies published in English. The search in the original review retrieved 740 articles, of which 523 were excluded based on the title. Of the remaining 217 articles, 122 were excluded after reading the abstract, and 58 after reading the full text and taking into account the selection criteria specified in Table 2. This left 30 articles: Seven on MetS, nine on obesity, four on T2DM, and 10 on CVD [6] (Figure 2).

## 3. Results

The new search retrieved 318 articles, of which 186 were excluded on screening the title. Of the remaining 132 articles, 69 were excluded after reading the abstract, and 43 after reading the full text, leaving 20 articles: Five on MetS, five on obesity, three on T2DM, and seven on CVD (Figure 2). Thus, the total number of articles included in the current review was 50: 30 from the original review and 20 from the updated one. Of these, 12 were on MetS, 14 on obesity, seven on T2DM, and 17 on CVD (Table 4 and Table 5). The five CQs were addressed based on different levels of scientific evidence. The level of evidence for each question is shown below, together with the corresponding rationale.

### 3.1. CQ 1: What Effect Does the MedDiet Have on Weight Reduction in Overweight and Obese Patients?

Level of evidence: High

• **MedDiet adherence reduces obesity and abdominal adiposity**

*Rationale:* A reduction in obesity and abdominal adiposity is a well-reported effect of the MedDiet in controlled clinical trials and prospective studies. A small study in Italy showed that obese women with greater adherence to a moderately hypocaloric MedDiet experienced a significant reduction in weight and BMI (body mass index) [19]. A subsequent Croatian dietary intervention study showed that higher MedDiet adherence favored greater weight loss in obese people than a low-fat diet [20]. In Spain, several sub-studies within the PREDIMED (PREvención con DIeta MEDiterránea [Prevention with a Mediterranean Diet]) clinical trial have shown that MedDiet adherence is associated with a lower level of inflammation [21] and a greater reduction in total weight, WC (waist circumference), BMI (especially for those following a MedDiet supplemented with extra-virgin olive oil) [22,23], and blood pressure, as well as increased levels of high-density lipoprotein cholesterol (HDL-C) [24].

• **The MedDiet reduces CVD incidence and mortality**

*Rationale:* Until just a few years ago, the American Heart Association recommended a low-fat diet in overweight patients with high cardiovascular risk [25]. Data, however, from the PREDIMED cohort, widely demonstrated the superiority of the MedDiet in reducing CVD risk and mortality in individuals without CVD but with other baseline risk factors [26]. A systematic review by Nissensohn et al. [27] of the effects of the MedDiet and a low-fat diet also found evidence of an association between the MedDiet and a reduction in systolic and diastolic blood pressure. Higher MedDiet adherence has also been linked to a reduction in CVD risk and mortality in the general population (including obese and non-obese individuals) without CVD [28,29,30,31,32,33,34,35,36,37,38,39].

### 3.2. CQ 2: What Effect Does the MedDiet Have on the Incidence and Prevention of T2DM?

Level of evidence: Moderate

• **The MedDiet reduces the incidence of T2DM in healthy individuals**

*Rationale:* Several studies have shown a reduction in the risk of de novo T2DM in healthy individuals with high MedDiet adherence [40,41,42,43]. By contrast, a meta-analysis found no evidence of differences between the MedDiet and control diets in terms of their effect on the risk of T2DM in non-diabetic individuals [44]. Abiemo et al. [45] studied the effect of the MedDiet in the general population and found that it reduced glucose and insulin levels, but not the incidence of T2DM in non-diabetics.

• **The MedDiet reduces the symptoms of T2DM and modulates disease course**

*Rationale:* MedDiet adherence has been found to reduce glycated hemoglobin (HbA1c) [43,44], CRP (C-reactive protein), and adiponectin [46] levels in diabetic patients.

### 3.3. CQ 3: What Effect Does the MedDiet Have on Established MetS or on the Risk of Developing MetS?

Level of evidence: Moderate-to-high

• **High MedDiet adherence decreases some risk factors for MetS**

*Rationale:* MedDiet adherence, compared against a control diet, has been found to reduce WC and blood pressure, and to increase HDL-C levels [24,47]. Other studies have found no association between the MedDiet and the prevalence of MetS, although some components of the diet showed a protective effect on MetS and its components [48]. 

• **The MedDiet reduces some risk factors for MetS in healthy individuals**

*Rationale:* Steffen et al. [49] showed that the incidence of MetS in healthy individuals was inversely proportional to the level of MedDiet adherence. Individuals with lower adherence showed greater abdominal adiposity and a higher percentage of low HDL-C levels over a period of 25 years. Similarly, Rumawas et al. [50] reported that individuals with greater MedDiet adherence had a significantly lower incidence of MetS in addition to lower WC and triglyceride levels, and higher HDL-C levels after 7 years of follow-up. These findings were corroborated by Kess-Guyot et al. [51]. Mirmiran et al. [52], in turn, in a prospective analysis of the MedDiet modified for the Iranian population that took into account the ratio of monounsaturated fatty acids (MUFAs) to polyunsaturated fatty acids (PUFAs) instead of olive oil consumption, found no evidence of an association between MedDiet adherence and incidence of MetS. Another prospective study by Alvarez-Leon et al. [48], which also studied the MUFA/PUFA ratio rather than olive oil consumption, also found no association between MedDiet adherence and incidence of MetS. 

### 3.4. CQ 4: What Effect Does the MedDiet Have on the Prevention of CVD and the Modulation of Disease Course?

Level of evidence: High

• **MedDiet adherence reduces the incidence of CVD in individuals with high cardiovascular risk**

*Rationale:* The protective role of the MedDiet on the incidence of cardiovascular events has been widely demonstrated in large clinical trials [28,30,35,37,39,53,54,55]. Furthermore, Estruch et al. [26] showed that, compared against the low-fat diet recommended by the American Heart Association, the MedDiet supplemented with nuts or extra-virgin olive oil protected high-risk individuals from CVD. A meta-analysis of 20 studies containing data from 888,257 individuals by Grosso et al. [56] showed that increased MedDiet adherence was associated with a relative risk reduction of 40% for CVD incidence.

• **MedDiet adherence reduces CVD mortality in individuals without CVD but with high cardiovascular risk**

*Rationale:* Several studies have reported an association between the MedDiet and a reduction in CVD mortality in individuals with risk factors for CVD [37,38,39]. Bonaccio et al. [38], in turn, showed that high MedDiet adherence was associated with a relative risk reduction of 34% for CVD mortality in patients with T2DM.

• **MedDiet adherence reduces CVD incidence and mortality in the general population**

*Rationale:* MedDiet intervention studies in the general population without baseline data on health status have concluded that adherence to the diet has a protective effect on CVD incidence and mortality [31,32,33,36,37,57,58]. A study by Fung et al. [32] of women with T2DM without a history of CVD also showed that high MedDiet adherence protected against CVD risk and associated mortality. Similarly, Knoop et al. [59] showed that higher MedDiet adherence was associated with reduced CVD-specific and all-cause mortality in the elderly population (70–90 years).

### 3.5. CQ 5: What Effect Does the MedDiet Have on Weight Gain and Abdominal Adiposity in Healthy Individuals and Individuals Without Overweight?

Level of evidence: Moderate-to-high

• **MedDiet adherence decreases weight gain and BMI in the general population**

*Rationale:*Several studies have shown that high MedDiet adherence reduces weight gain and BMI in the general population in the long term [60,61,62]. An additional study by Paletas et al. [63] reported that MedDiet adherence contributed to weight control in individuals who were overweight at baseline. This effect was not observed for those with normal weight.

• **MedDiet adherence reduces WC in the general population**

*Rationale:* Studies by Rumawas et al. [50] and Steffen et al. [49] showed that individuals with high MedDiet adherence have a smaller WC. Romaguera et al. [64] also observed this finding in individuals with a BMI between 20.09 and 20.17.

## 4. Discussion

The MedDiet is a well-known, prudent dietary pattern with health benefits supported by an exponentially increasing wealth of scientific evidence. Based on the most recent and accurate scientific evidence available, the aim of this review was to shed light on the therapeutic and preventive effects of the MedDiet on diseases encompassed by the umbrella term *cardiodiabesity*, in order to inform and guide the development of CPGs for physicians and health professionals. The review addressed five CQs containing key PICO components. The evidence for CQ 1 on the association between the MedDiet and improved health in overweight and obese individuals indicates that it is precisely this population that would benefit the most from the weight loss associated with the MedDiet, and from the additional benefits of a lower risk of CVD incidence and mortality. The evidence for CQ 2, regarding the potential effects of the MedDiet on T2DM incidence and prevention, was moderate. Although some studies provided solid evidence of an effect [40,41,42,43], there were discrepancies in relation to the impact of high MedDiet adherence on the risk of T2DM in healthy individuals, and to the reduction of symptoms in those who already had the disease. However, it should be noted that fewer studies have been conducted on the effect of the MedDiet—either through an intervention or simply by measuring adherence to it—on T2DM prevention or amelioration than on other cardiodiabesity outcomes. The evidence for CQ 3, on the association between the MedDiet and MetS, showed that, overall, high adherence was related to decreased risk factors for MetS. The level of evidence was moderate-to-high, although only two studies provided clear evidence of a protective effect in MetS patients. The findings relating to the risk of MetS in healthy individuals were conflicting. The answers to CQ 1 and CQ 4 indicated that overweight and obese individuals who adhered to the MedDiet were most likely to benefit from CVD prevention and disease course modulation. A reduction in CVD incidence and mortality was observed for high-risk individuals and the general population. Considering that CVD is one of the leading causes of morbidity and mortality in Western countries, a medium-term reduction in its incidence would be one of the main benefits of promoting MedDiet adherence. CQ 5 addressed the effect of the MedDiet on weight in non-overweight and non-obese individuals. The resulting evidence, graded as moderate-to-high, indicated an inverse relationship between MedDiet adherence and an increase in weight, BMI, and WC.

The evidence used to answer the five CQs was found in 50 articles from the scientific and medical literature on the association between the MedDiet and cardiodiabesity. Twenty of the articles were new and 30 were from the previous review on this topic [6], which found strong evidence of the beneficial effects of MedDiet adherence in patients with CVD, T2DM, MetS, and obesity [6]. From the 37 articles included in the previous paper [6], seven were excluded as they did not meet the inclusion criteria defined in this analysis. Taken together, the 50 articles show strong evidence that the MedDiet plays therapeutic and preventive roles in cardiodiabesity. There was a high level of evidence showing that MedDiet adherence improves the health of overweight and obese patients by reducing weight and WC, and lowering CVD incidence and mortality [19,20,21,22,23,24,25,26,27,28,29,30,31,32,33,34,35,36,37,38,39]. Moderate evidence of a preventive effect of the MedDiet on T2DM was found for patients with the disease, and in individuals with and without risk factors [40,41,42,43,44,45,46]. Evidence of the preventive and therapeutic roles of MedDiet adherence was moderate-to-high for MetS [24,47,48,49,50,51,52] and high for CVD risk. Individuals with risk factors and the general population benefited from a reduction in CVD incidence and mortality [28,30,31,32,33,35,36,37,38,39,53,54,55,56,57,58,59], thus supporting previous meta-analysis findings [57]. The association between MedDiet adherence and low weight gain, BMI, and WC in non-obese individuals was supported by low-to-high evidence [49,50,60,61,62,63,64]. Many mechanisms underlying the beneficial effects of the MedDiet have been described elsewhere and are mainly related to improvements in lipid profile, oxidative stress, inflammation status, glucose metabolism, vascular integrity, and effects on hormone status and gut microbiota-mediated metabolic health, amongst others [4]. 

Regarding its beneficial effects on cardiovascular health, higher MedDiet adherence has been shown to improve complex processes relating to atherosclerosis, such as the atherogenicity of LDLs or the functionality of HDLs. In the latter case, this is particularly so when the MedDiet is supplemented with virgin olive oil. Epidemiological trials from the 1960s suggested that MedDiet adherence was associated with decreased rates of CVD. Many studies have demonstrated a mortality benefit from a Mediterranean or Mediterranean-like diet after MI. One example is the Lyon Diet Heart Study [67], which showed that a MedDiet reduced recurrent cardiovascular events by 50%–70% among MI patients. However, the role of the MedDiet in the primary prevention of CVD had not been well established until the PREDIMED trial. That trial randomized 7447 Spanish patients at high risk for CVD to one of three diets: (1) A MedDiet supplemented with extra-virgin olive oil; (2) a MedDiet supplemented with nuts; or (3) a control diet encouraging the intake of low-fat foodstuffs [26]. In both MedDiet groups, there was a statistically significant reduction in the rate of the composite primary outcome of MI, stroke, or cardiovascular death after more than four years. Regarding the strength of the association, there was a 30% absolute reduction in the incidence of major CVD events in the MedDiet groups. These findings were consistent with the previous large body of observational scientific evidence, and potential confounders as alternative explanations were discarded. A dose–response gradient was observed, whereby greater MedDiet adherence showed increased protection. Every two-point increase in adherence was associated with a 25% reduction in CVD. The results were consistent with the known facts and accepted paradigms for the natural history and biology of CVD. The beneficial effects on surrogate markers of CVD risk added to the consistency. The epidemiological evidence of low CHD rates in Mediterranean countries also supports a protective effect of the MedDiet. Regarding experimental evidence, the PREDIMED RCT was intensively scrutinized in 2018 after certain comments on the randomization procedures led to the study being retracted and republished by the same research group [26,68]. In the assessment of the quality of the RCT, inappropriate allocation concealment was among the reasons noted [69]. The authors performed several complicated analyses to attempt to control for these deficiencies, which all seemed to confirm the original findings of that study. Thus, the updated publication documented similar findings, but the authors were unable to confirm adherence to randomization schemes, given that documentation was missing (supplemental appendix of [26]). Other high-quality dietary patterns such as the Dietary Approaches to Stop Hypertension (DASH) diet [70] and the prudent healthy pattern measured by the Healthy Eating Index [71] have also been associated with a reduced incidence of CVD events. However, the evidence collected and analyzed for these patterns is not as robust as the evidence provided by PREDIMED. 

The aim of this study was to analyze the effects of the MedDiet as a diet and lifestyle, and not the effects of specific types of food. Studies focusing on the benefits of specific food groups in patients with cardiodiabesity were therefore excluded, even though some showed evidence of very strong preventive and therapeutic effects. Consumption of dairy fat, for instance, has been linked to a low incidence of MetS, while that of whole and skimmed cheese has been linked to a higher risk of MetS, and that of whole yogurt to a decrease in all the risk factors for MetS [72]. Indeed, while there have been concerns about a diet’s fat content since the last century, a number of reviews have shown that a low-fat diet is not effective at preventing cardiometabolic disease [73]. A direct association has also been observed between the consumption of tea and coffee and a lower incidence of MetS within the context of the MedDiet [74]. In another study of individuals with a moderate cardiovascular risk, moderate consumers of red wine had a lower risk of MetS than non-drinkers [75]. Interestingly, this effect was more pronounced in women. The consumption of sugary drinks has been linked to increased WC [76,77]. Mozaffarian et al. [78] conducted an in-depth review of the role of the main components of the MedDiet and other widely consumed foods in relation to health status. Their findings supported the widely agreed beneficial effects of fruit, nuts, fish, vegetables, vegetable oils, whole grains, beans, and yogurt, and the harmful effects of refined grains, starches, sugars, processed meats, high-sodium foods, and trans-fat foodstuffs. Foodstuffs for which there are still no proven beneficial or harmful effects include cheese, eggs, poultry, milk, butter, and unprocessed red meat. Albeit indirectly, the findings of this review show the benefits of the main components of the MedDiet. As called for by previous studies, the relative effects of specific food groups need further investigation [6,55,58,65,66].

Our study has some limitations. Although some of the studies analyzed in the review did not find a strong association between MedDiet adherence and the main outcomes analyzed, they did find positive links to intermediate risk factors. Certain discrepancies could be due to numerous aspects relating to the inherent limitations of the different studies. 

In addition, when analyzing the literature, it is necessary to be aware of the different confounders and indices to ensure appropriate quantitative and/or qualitative measures [79]. The likelihood of heterogeneous measures of cause and effect should also be taken into account when interpreting the association between the MedDiet and the different health outcomes, as has been done in two recent meta-analyses that corroborate the results of the PICO analysis [80]. The level of heterogeneity of the included studies is also our concern. 

This study is based on the 2014 study of the association between the MedDiet and cardiodiabesity by García-Fernández et al. [6]. The literature review was updated using the same search and a similar methodology to address a series of CQs based on scientific evidence on the association between the MedDiet and cardiodiabesity, with a view to informing future CPGs on how to treat and prevent obesity, MetS, T2DM, and CVD. The evidence uncovered provides solid support of an inverse relationship between MedDiet adherence and cardiodiabesity outcomes. 

## 5. Conclusions

Recent scientific evidence has shown that the MedDiet, which is listed as a UNESCO (United Nations Educational, Scientific and Cultural Organization) Intangible Cultural Heritage of Humanity [81,82] and referred to in the 2015–2020 American dietary guidelines [83] as an example of a healthy eating pattern, has a beneficial effect on health and sustainability. It also has an important social component [84,85]. The scientific basis for developing evidence-based CPGs consistent with international standards, such as those promoted by the Scottish Intercollegiate Guidelines Network (SIGN) and the United Kingdom’s National Institute for Health and Care Excellence (NICE), has been provided. The reviewed studies show strong evidence of an association between MedDiet adherence and outcomes in cardiodiabesity, which encompasses CVD, T2DM, MetS, and obesity. The MedDiet plays a role in obesity and MetS prevention in healthy or at-risk individuals, and in mortality risk reduction in overweight or obese individuals. Furthermore, it decreases the incidence of T2DM and CVD in healthy individuals, and reduces the severity of symptoms in individuals that already have those diseases. The scientific evidence seems to support the conclusion that MedDiet adherence is a preventive and therapeutic tool for cardiodiabesity.

## Figures and Tables

**Table 1:** Clinical questions (CQs) based on the Patient, Intervention, Comparison, and Outcome (PICO) method.

P: Who Are the Patients/Participants in the Study?	I: What Intervention Is Being Examined?	C: Against What is the Intervention of Interest Being Compared?	O: What Are the Measured Results (Outcomes)?	CQs
Men and women with overweight or obesity and/or MetS	Application of MedDiet and/or monitoring of MedDiet adherence	Epidemiologically similar control group that does not follow the MedDiet	Reduction in weight, BMI, and/or WC	**CQ 1:** What effect does the MedDiet have on weight reduction in overweight and obese patients?
Men and women with or at risk of T2DM	Application of MedDiet and/or monitoring of MedDiet adherence	Epidemiologically similar control group that does not follow the MedDiet	Reduction in risk of all-cause mortality and mortality due to CVD, heart attack, or T2DM	**CQ 2:** What effect does the MedDiet have on the incidence and prevention of T2DM?
Healthy men and women with MetS or risk factors for MetS	Application of MedDiet and/or monitoring of MedDiet adherence	Epidemiologically similar control group that does not follow the MedDiet	Reduction in incidence or severity of MetS	**CQ 3:** What effect does the MedDiet have on established MetS or on the risk of developing MetS?
Men and women	Application of MedDiet and/or monitoring of MedDiet adherence	Epidemiologically similar control group that does not follow the MedDiet	Reduction in CVD incidence or mortality	**CQ 4:** What effect does the MedDiet have on the prevention of CVD and the modulation of disease course?
Men and women	Application of MedDiet and/or monitoring of MedDiet adherence	Epidemiologically similar control group that does not follow the MedDiet	Reduction in weight gain, BMI, or WC	**CQ 5:** What effect does the MedDiet have on weight gain and abdominal adiposity in healthy individuals and individuals without overweight?

Abbreviations not previously defined: BMI, body mass index; WC, waist circumference.

**Table 2:** Study selection criteria.

Item	Inclusion Criteria	Exclusion Criteria
**Population**	Adults (>18 years old)	Children Experimental animal studies
**Intervention**	Dietary interventions with the pure MedDiet (defined in the study) or the MedDiet with reinforcement of one of the food components (e.g., olive oil or nuts) No intervention (analysis of MedDiet adherence [defined in the study])	Other food interventions and interventions involving specific foods even though they form part of the MedDiet. Other non-dietary interventions (e.g., pharmacological or surgical).
**Comparator**	Non-dietary intervention, prudent diet, Westernized diet, or any type of diet other than the MedDiet Non-adherence to the MedDiet	
**Results (outcomes)**	Weight reduction measured as weight (kg, lb, %), WC, hip-waist ratio, percentage of body fat, maintenance of weight loss Cardiovascular events: MI, heart failure, hospitalization for MI or heart failure Cardiovascular risk factors: total cholesterol factors, HDL-C, LDL-C, non-HDL-C, triglycerides, diabetes, smoking, CRP Morbidity: cardiovascular damage, chronic renal failure, non-alcoholic steatohepatitis, depression Mortality: CVD, all causes Changes in body composition: improved quality of life, functionality, disability	Self-reported weight
**Time**	No time limits Minimum 6-month follow-up	Fewer than 6 months of follow-up *
**Study design**	Systematic reviews and clinical trials	Other
**Language**	English	Other (despite availability of an English abstract)
**Publication type**	Systematic reviews and meta-analyses	Other
**Publication date**	From October 2013 to July 2016	All others

Abbreviations not previously defined: CRP, C-reactive protein; HDL-C, high-density lipoprotein cholesterol; LDL-C, low-density lipoprotein cholesterol; MI, myocardial infarction. * Not excluded, but assigned a lower level of evidence.

**Table 3:** Levels of scientific evidence.

Study Characteristics	Level of Evidence
• Well-designed, well-executed RCTs with assessment of health outcomes, representative of the populations to which the results apply • Meta-analyses of the aforementioned RCTs • High level of certainty about the estimated effects and very little likelihood that more research on the subject would alter this certainty	High
• RCTs with minor limitations affecting applicability of or confidence in the results • Meta-analysis of the aforementioned RCTs • Moderate certainty about the estimated effects and likelihood that more research on the subject would alter this certainty	Moderate
• RCTs with major limitations • Non-RCTs and observational studies with major constraints affecting applicability of or confidence in the results • Uncontrolled clinical studies without an adequate comparison group • Psychological studies in humans and meta-analyses of these • Low certainty about the estimated effects and strong likelihood that more research on the subject would alter this certainty	Low

Abbreviations not previously defined: RCT, randomized controlled trial.

**Table 4:** Articles included in this review published between October 2013 and July 2016.

Author, Year	Type of Study	Country	Sex, Age (y) and Number of Participants	Initial Disease	Follow-Up (y)	Components of MedDiet Index	Object of Study	Results	Confounders
Ruiz-Canela et al., 2015 [21]	Multicenter parallel-group RCT (PREDIMED)	Spain	3111 men (ages 55–80) 4125 women (ages 60–80) N total = 7236	No CVD or T2DM but three risk factors for CVD: smoking, hypertension, high LDL-C, low HDL-C, BMI ≥ 25 kg/m^2^, family history of premature CVD	-	PREDIMED [26]	Obesity	Adjusted difference in WHtR for women and men between the highest and lowest quintiles of DII: 1.60% (95% CI, 0.87–2.33) and 1.04% (95% CI, 0.35–1.74), respectively	
Nissensohn et al., 2015 [27]	Systematic review and meta-analysis	Spain	Men and women (age not specified) N total > 7000	Different depending on the study	>2	Different depending on the study	Cardiodiabesity	MedDiet vs. low-fat diet: decrease in systolic and diastolic blood pressure	
Eguaras et al., 2015 [54]	RCT (PREDIMED)	Spain	3241 men (ages 55–80) 4297 women (ages 60–80) N total = 7538	High risk of CVD due to T2DM or presence of three risk factors for CVD	4.8	PREDIMED [26]	Obesity and CVD	Increased risk of CVD events was apparent for the highest vs. the lowest quartiles of WHtR (HR, 1.98; 95% CI, 1.10–3.57; linear trend: *p* = 0.019) only in control diet group	Age, sex, multivariate
Hadziabdic et al., 2016 [20]	Parallel-group RCT	Croatia	Men and women (ages 18–69) N total = 84	Obesity (≥30 kg/m^2^)	1	(+) vegetables, fruit, whole grains, (-) red meat (+) fish and poultry. 1573 kcal/day 33 g of olive oil/day and 56 g of nuts/week	Obesity	MedDiet vs. low-fat diet: tendency towards high weight loss (kg)	
Alvarez-Perez et al., 2016 [22]	Multicenter, parallel-group RCT (PREDIMED)	Spain	Men (ages 55–80) and women (ages 60–80) N total = 305	No CVD or T2DM but three risk factors for CVD: smoking, hypertension, high LDL-C, low HDL-C, overweight/obesity, family history of premature CVD	1	PREDIMED [26]	Obesity	Low-fat diet decreased total body weight but increased total body fat. MedDiet + nuts decreased total body weight. MedDiet + extra-virgin olive oil decreased total body weight, BMI, and WC.	Sex and age
Casas et al., 2014 [55]	Parallel-group RCT (PREDIMED)	Spain	77 men and 87 women (average age 67.7) N total = 164	No CVD or T2DM but three risk factors for CVD: smoking, hypertension, high LDL-C, low HDL-C, BMI ≥ 25 kg/m^2^, family history of premature CVD	1	PREDIMED [26]	CVD	MedDiet reduced systolic (*p* = 0.02) and diastolic (*p* = 0.02) blood pressure, total cholesterol (*p* = 0.04) and LDL-C by 5–9% (*p* = 0.04). MedDiet significantly reduced inflammatory markers (e.g., VCAM and ICAM) and adhesion molecules (e.g., CD40).	
Grosso et al., 2015 [56]	Systematic review and meta-analysis (20 studies)	Several	Men and women (ages 20–70) N total = 888,257	Established CVD, risk factors for CVD, elderly	-	MedDiet	CVD	Higher MedDiet adherence was associated with a 40% relative risk reduction in CVD incidence and mortality. Reduced CVD risk for consumption of olive oil, vegetables, fruit, and pulses, and increased CVD risk for consumption of dairy products. No difference for consumption of fish, alcohol, cereals, or red meat.	
Bonaccio et al., 2014 [38]	Cohort study	Italy	139 men and 643 women (average age 62.6) N total = 1995	T2DM at the beginning of the study	4	EPIC-Trichopoulou score [65]	CVD and mortality	Higher MedDiet adherence was associated with a 37% relative risk reduction in CVD mortality and a 34% relative risk reduction in cerebrovascular-event mortality. Adherence to consumption of vegetables and olive oil reduced mortality by 21%. A reduction was observed only when CVD mortality was considered (HR, 0.66; 0.46–0.95). The MedDiet was associated with a reduced risk of death overall (HR, 0.81; 0.62–1.07).	Age, sex, education, oil intake, blood glucose
Menotti 2015 [59]	Prospective study of MedDiet adherence and lifestyle in Seven Countries CVD study	Italy	Men and women (age up to 90) N total = 1677	General rural population	≤50	MedDiet: 18 food groups [29]	CVD	MedDiet adherence was associated with lower CVD incidence. Cox proportional HRs for CHD: 1.45 (95%, CI, 1.11–1.90) for heavy smokers vs. non-smokers; 0.67 (95% CI, 0.50–0.89) for vigorous activity vs. sedentary habits, and 0.62 (95% CI, 0.47–0.83) for MedDiet vs. non-MedDiet.	Smokers and physical activity
Stefler et al., 2015 [39]	Prospective study of HAPIEE cohort	Poland, Russia, and Czech Republic	8787 men and 10,546 women (age not specified) N total = 19,333	Absence of CVD and diabetes	7	MedDiet recommendations [66] with categorization of 17 points	CVD	One SD increase in MDS inversely associated with all-cause mortality (HR, 0.93; 95% CI, 0.88–0.98) and CVD (HR, 0.90; 95% CI, 0.81–0.99). Inverse but non-significant link found for CHD (HR, 0.90; 95% CI, 0.78–1.03) and stroke (HR, 0.87; 95% CI, 0.71–1.07).	
Turati et al., 2015 [36]	Prospective cohort study (EPIC)	Greece	8246 men and 12,029 women (ages 20–86) N total = 20,275	Absence of CVD, cancer, and diabetes	10.4	MedDiet defined according to Trichopoulou [65]	CVD	Significant positive association between glycemic load and CHD incidence (HR for highest vs. lowest tertiles, 1.41; 95% CI, 1.05–1.90). High MedDiet adherence with low/moderate glycemic load associated with lower risk of CHD incidence (HR, 0.61; 95% CI, 0.39–0.95) and mortality (HR, 0.47; 95% CI, 0.23–96).	Sex, BMI
Stewart et al., 2016 [37]	RCT	30 countries	12,556 men and 2926 women (average age 64.2) N total = 15,482	Previous MI with a risk factor: > 60 years, DM under treatment, HDL-C < 1.03 mmol/L, smoker or ex-smoker, glomerular filtration rate > 30 < 60 mL/min or albuminuria or polyvascular disease	3.7	MedDiet defined according to Turati [36]. Eggs and dairy products not included.	CVD	MedDiet adherence (MDS > 12) associated with lower CVD incidence and mortality. One-unit increase in MDS > 12 associated with lower MACE after adjusting for all covariates (+1 category HR, 0.95; 95% CI, 0.91– 0.98, *p* = 0.002). No association between Western diet score (adjusted model +1 category HR, 0.99; 95% CI, 0.97– 1.01) and MACE.	Geography, education
Esposito et al., 2015 [43]	Systematic review and meta-analysis	Several	Men and women (age not specified) N total = 1266	Overweight or obesity with T2DM	>0.5	MedDiet defined according to PREDIMED [26]	T2DM	Higher MedDiet adherence lowered HbA1c. MedDiet reduced incidence of T2DM.	
Sleiman et al., [44]	Systematic review	Several	Men and women (age not specified) N total = 1266	Obesity with T2DM and non-high-risk diabetes	0.5–2	Different depending on study	T2DM	Fasting glucose increased and HbA1c decreased in individuals following the MedDiet. No differences for MedDiet and control diet in non-diabetic patients.	
Maiorino et al., 2016 [46]	Parallel-group RCT (MEDITA)	Italy	Men and women (age not specified) N total = 215	Recent diagnosis of T2DM	8.1	MedDiet	T2DM	MedDiet decreased CRP and increased adiponectin	
Babio et al., 2014 [23]	Multicenter, parallel-group RCT (PREDIMED)	Spain	2437 men (ages 55–80) and 3364 women (ages 60–80) N total = 5801	No CVD or T2DM but three risk factors for CVD: smoking, hypertension, high LDL-C, low HDL-C, BMI ≥ 25 kg/m^2^, family history of premature CVD	4.8	PREDIMED [26]	MetS	The risk of MetS was higher in MedDiet vs. control diet (control vs. olive oil: HR, 1.10; 95% CI, 0.94–1.30, *p* = 0.231; control vs. nuts: HR, 1.08; 95% CI 0.92–1.27, *p* = 0.3). Compared against control group, participants on either MedDiet were more likely to undergo reversion (control vs. olive oil: HR, 1.35; 95% CI: 1.15–1.58, *p* < 0.001; control vs. nuts: HR, 1.28; 95% CI, 1.08–1.51; *p* < 0.001).	
Steffen et al., 2014 [49]	Prospective study of MedDiet adherence and CVD (CARDIA)	USA	2140 men and 2573 women (ages 18–30 at the beginning of the study) N total = 4713	Absence of MetS	25	Modified by Trichopoulou [65]	MetS	Incidence of MetS inversely proportional to MedDiet adherence. Lower adherence → higher abdominal adiposity and % low HDL-C. The HRs and 95% CI from category 1 to category 5 were 1.0; 0.94 (0.76, 1.15); 0.84 (0.68, 1.04); 0.73 (0.58, 0.92); and 0.72 (0.54, 0.96), respectively (*p* = 0.005).	Age, education, physical activity, and race
Gomez-Huelgas 2015 [24]	Cross-sectional study to determine prevalence of MetS	Spain	55.1% men and 44.9% women (average age 53.8) N total = 406	MetS as defined by the International Society of Diabetes	3	MedDiet (14 points) according to PREDIMED	MetS	MedDiet → greater decrease in WC and blood pressure and higher HDL than the control group.	
Mirmiran et al., 2015 [52]	Prospective study to identify and prevent non-communicable diseases	Iran	44.8% men and 55.2% women (average age 39.1) N total = 2241	Healthy individuals without T2DM or MetS	3	MedDiet defined according to Trichopoulou [65]. MUFA/PUFA ratio; no olive oil intake	MetS	In the multivariable model, the adjusted odds ratio (OR) for developing MetS did not differ significantly between participants in the highest MDS tertile (OR, 0.88; 95% CI, 0.62–1.23) or Sofi-MDS (OR, 1.12; 95% CI, 0.77–1.62) and those in the lowest tertiles.	Age, sex, intake, physical activity, smoker, BMI
Kastorini et al., 2016 [35]	ATTICA	Greece	50% men and 50% women (ages 18–89) N total = 2020	Absence of CVD	8.41	MedDiet	MetS and CVD	10% increase in MedDiet adherence associated with 15% less probability of developing CVD. Individuals with low MedDiet adherence were twice as likely to develop MetS. MetS associated with two-fold increased odds of CVD incidence (OR, 2.04; 95% CI, 1.31–3.17) in participants with low MedDiet adherence.	Age, sex, family history, smoker, history of MetS

Abbreviations not previously defined: DII, dietary inflammatory index; HR, hazard ratio; CHD, coronary heart disease; ICAM, intercellular adhesion molecule; MACE, major adverse cardiovascular events; MDS, Mediterranean diet score; VCAM, vascular cell adhesion molecule; WHtR, waist to height ratio.

**Table 5:** Scientific evidence for health outcomes related to the key CQs on the Mediterranean Diet (MedDiet).

CQs	Scientific Evidence	References
CQ 1: What effect does the MedDiet have on weight reduction in overweight and obese patients?	MedDiet adherence reduces obesity and abdominal adiposity.	Andreoli et al., 2008 [19]; Hadžiabdić et al., 2015 [20]; Ruiz-Canela et al., 2015 [21]; Álvarez Pérez et al., 2016 [22]; Babio et al., 2016 [23]; Gómez-Huelgas et al., 2015 [24].
	The MedDiet reduces CVD incidence and mortality.	US Department of Health and Human Services et al., 1980 [25]; Estruch et al., 2013 [26]; Nissensohn et al., 2016 [27]; Martínez-Gónzalez et al., 2011 [28]; Menotti et al., 2012 [29]; Gullar-Castillón et al., 2012 [30]; Gardener et al., 2011 [31]; Fung et al., 2009 [32]; Buckland et al., 2009 [33]; Trichopoulou et al., 2005 [34]; Kastorini et al., 2016 [35]; Turati et al., 2015 [36]; Stewart et al., 2016 [37]; Bonaccio et al., 2014 [38]; Stefler et al., 2015 [39].
CQ 2: What effect does the MedDiet have on the incidence and prevention of T2DM?	The MedDiet reduces the incidence of T2DM in healthy individuals.	Panagiotakos et al., 2005 [40]; Martínez-González et al., 2008 [41]; Salas-Salvado et al., 2011 [42]; Esposito et al., 2015 [43]; Sleiman et al., 2015 [44]; Abiemo et al., 2012 [45].
	The MedDiet reduces the symptoms of T2DM and modulates disease course.	Esposito et al., 2015 [43]; Sleiman et al., 2015 [44]; Maiorino et al., 2016 [46].
CQ 3: What effect does the MedDiet have on established MetS or on the risk of developing MetS?	High MedDiet adherence reduces some of the risk factors for MetS in patients with the disease.	Gómez-Huelgas et al., 2015 [24]; Salas-Salvado et al., 2013 [47]; Alvarez Leon et al., 2006 [48].
	The MedDiet reduces some of the risk factors for MetS in healthy individuals.	Alvarez Leon et al., 2006 [48]; Steffen et al., 2014 [49]; Rumawas et al., 2009 [50]; Kesse-Guyot et al., 2013 [51]; Mirmiran et al., 2015 [52].
CQ 4: What effect does the MedDiet have on the prevention of CVD and the modulation of disease course?	MedDiet adherence reduces the incidence of CVD in individuals with high cardiovascular risk.	Martínez-González et al., 2011 [28]; Gullar-Castillón et al., 2012 [30]; Kastorini et al., 2016 [35]; Stewart et al., 2016 [37]; Stefler et al., 2015 [39]; Panagiotakos et al., 2007 [53]; Eguaras et al., 2015 [54]; Casas et al., 2014 [55]; Estruch et al., 2013 [26]; Grosso et al., 2015 [56].
	MedDiet adherence reduces CVD mortality in individuals without CVD but with high cardiovascular risk.	Stewart et al., 2016 [37]; Bonaccio et al., 2014 [38]; Stefler et al., 2015 [39].
	MedDiet adherence reduces CVD incidence and mortality in the general population.	Gardener et al., 2011 [31]; Fung et al., 2009 [32]; Buckland et al., 2009 [33]; Turati et al., 2015 [36]; Stewart et al., 2016 [37]; Menotti et al., 2012 [57]; Menotti, 2015 [58]; Knoops et al., 2004 [59].
CQ 5: What effect does the MedDiet have on weight gain and abdominal adiposity in healthy individuals and individuals without overweight?	MedDiet adherence decreases weight gain and/or BMI in the general population.	Romaguera et al., 2010 [60]; Schröder et al., 2004 [61]; Goulet et al., 2003 [62]; Paletas et al., 2010 [63].
	MedDiet adherence reduces WC in the general population.	Rumawas et al., 2009 [50]; Steffen et al., 2014 [49]; Romaguera et al., 2009 [64].
